# Accuracy of genomic selection in simulated populations mimicking the extent of linkage disequilibrium in beef cattle

**DOI:** 10.1186/1471-2156-12-80

**Published:** 2011-09-20

**Authors:** Fernanda V Brito, José Braccini Neto, Mehdi Sargolzaei, Jaime A Cobuci, Flavio S Schenkel

**Affiliations:** 1 Centre for Genetic Improvement of Livestock, University of Guelph, Guelph, ON N1G2W1, Canada; 2 Departamento de Zootecnia, Universidade Federal do Rio Grande do Sul, Caixa Postal 15100, Porto Alegre, RS, 91540-000, Brasil; 3 L'Alliance Boviteq, Saint-Hyacinthe, QC, J2T5H1, Canada

## Abstract

**Background:**

The success of genomic selection depends mainly on the extent of linkage disequilibrium (LD) between markers and quantitative trait loci (QTL), the number of animals in the training set (TS) and the heritability (h^2^) of the trait. The extent of LD depends on the genetic structure of the population and the density of markers. The aim of this study was to calculate accuracy of direct genomic estimated breeding values (DGEBV) using best linear unbiased genomic prediction (GBLUP) for different marker densities, heritabilities and sizes of the TS in simulated populations that mimicked previously reported extent and pattern of LD in beef cattle.

**Results:**

The accuracy of DGEBV increased significantly (p < 0.05) with the increase in the number of bulls in the TS (480, 960 or 1920), trait h^2 ^(0.10, 0.25 or 0.40) and marker densities (40 k or 800 k). Increasing the number of animals in the TS by 4-fold and using their phenotypes to estimate marker effects was not sufficient to maintain or increase the accuracy of DGEBV obtained using estimated breeding values (EBVs) when the trait h^2 ^was lower than 0.40 for both marker densities. Comparing to expected accuracies of parent average (PA), the gains by using DGEBV would be of 27%, 13% and 10% for trait h^2 ^equal to 0.10, 0.25 and 0.40, respectively, considering the scenario with 40 k markers and 1920 bulls in TS.

**Conclusions:**

As reported in dairy cattle, the size of the TS and the extent of LD have major impact on the accuracy of DGEBV. Based on the findings of this simulation study, large TS, as well as dense marker panels, aiming to increase the level of LD between markers and QTL, will likely be needed in beef cattle for successful implementation of genomic selection.

## Background

Genomic selection is a method of marker-assisted selection based on LD that potentially explores all QTL in the genome [1]. The breeding value (BV) is estimated by the sum of the effects of marker alleles or haplotypes covering the entire genome and its accuracy could be as high as 0.85 [1].

The BV calculated from the estimated effects of markers is often called DGEBV and the blended value between DGEBV and traditional EBV is often called Genomic Estimated Breeding Value (GEBV). The accuracy of DGEBV and GEBV depends on: 1) the level of LD between markers and QTL; 2) the number of animals in the TS; 3) the heritability of the trait and 4) the distribution of QTL effects [2].

Since 2009, national evaluations of Holstein bulls are being performed in the USA and Canada based on GEBV. Before that, a comprehensive study evaluating the reliability of GEBV of many economic traits in thousands of Holstein bulls genotyped with the Illumina BovineSNP50 Genotyping BeadChip (Illumina, Inc.,San Diego, California, USA) demonstrated that genomic PA of young animals were substantially higher than the reliability of traditional PA [3,4].

In beef cattle, the situation is quite different with respect to feasibility of genomic selection. There are several reasons for this, which include the genetic structure of herds, which involve a greater number of breeds with different origin and history of selection, resulting in significant differences in genetic parameters, such as LD and effective population size (N_e_), which influence the accuracy of genomic selection [5-7]. Furthermore, much of the impetus for using genomics in beef cattle is to allow selection for traits that are not routinely recorded. Simulation studies are still more frequent than studies with real beef cattle data, despite the availability of databases, mainly in *bos taurus *breeds [e.g., [8]]. However, there still exists a lack of data available for several beef cattle breeds, especially for indicine (zebu) breeds. The aim of this study was to evaluate the potential accuracy of DGEBV for two marker densities, different heritability levels and different sizes of TS in simulated populations that mimicked previously reported extent and pattern of LD in beef cattle.

## Methods

### Simulation

#### Population structure

Using the QMSim software [9], populations were simulated based on forward-in-time process [10] with either 40 k or 800 k single nucleotide polymorphism (SNP) markers, and 750 QTL across the 29 *bos taurus *autosomes (BTAs). In the first simulation step, 1000 generations with a constant size of 1000 were simulated, followed by 1020 generations with a gradual decrease in population size from 1000 to 200 in order to create initial LD and establish mutation-drift equilibrium in historical generations. The number of individuals of each sex remained the same in this step and the mating system was based on random union of gametes, randomly sampled from both the male and female gamete pools. Therefore only two evolutionary forces were considered in this step: mutation and drift. In the second step, an expansion of the population was created by initially randomly selecting 100 founder males and 100 founder females from the last generation of the historical population. In order to enlarge the population, eight generations were simulated with five offspring per dam and an exponential growth of the number of dams. The mating was again based on the random union of gametes and no selection was also considered in this step. In the case of simulating 800 k SNP markers, the expansion of the population was carried over six generations, instead of eight, due to computer time and memory requirements which resulted in a smaller population size.

In the next simulation step, two recent generation sets were simulated by selecting 640 males and 32000 females (POP1) or 160 males and 8000 females (POP2) from the last generation of the two expanded populations with 40 k or 800 k SNP markers, respectively. Then ten generations were simulated. Generations three to eight were used as TS and EBVs for this group were estimated by excluding information for generations nine and ten. The prediction set for both POP1 and POP2 included only animals born in generation ten.

The parameters used in the recent generations mimicked more closely to a real production system with one progeny per dam per year, 50% of male progeny, selection for high values of EBV and culling for low values of EBV with a replacement rate of 60% for sires and 20% for dams. Sires and dams were randomly mated.

**Figure 1:**
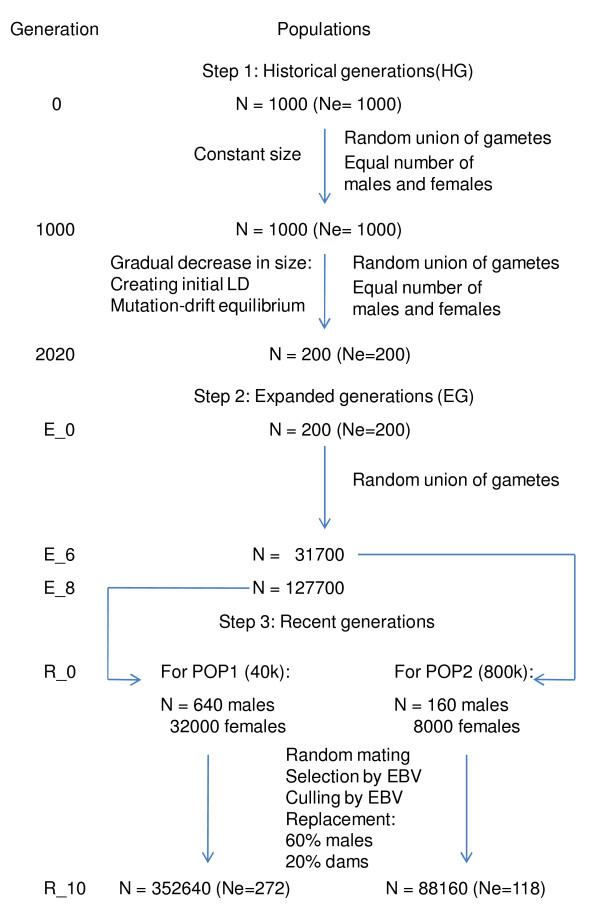
**Schematic representation of the simulation steps**.

The breeding values were estimated by BLUP, using Henderson's mixed linear equations [11] for an individual animal model, considering the true additive genetic variance. The rate of missing sire and dam information was 5%. A single trait with heritability of 0.10, 0.25 or 0.40 and phenotypic variance of 1.0 was simulated. The true breeding value of an individual was equal to the sum of the QTL additive effects and the phenotypes were generated by adding random residuals to the true breeding values. The whole simulation process was repeated 10 times. The parameters of simulation process are summarized in Table 1, while Figure 1 depicts the simulation steps.

**Table 1:** Parameters of the simulation process

Population structure	POP1/POP2
**Step 1: Historical generations (HG)**	
Number of generations(size) - phase 1	1000(1000)
Number of generations(size) - phase 2	1020(200)
	
**Step 2: Expanded generations (EG)**	
Number of founder males from HG	100
Number of founder females from HG	100
Number of generations	8/6
Number of offspring per dam	5
	
**Step 3: Recent generations**	
Number of founder males from EG	640/160
Number of founder females from EG	32000/8000
Number of generations	10
Number of offspring per dam	1
Ratio of male	50%
Mating system	Random
Replacement ratio for males	60%
Replacement ratio for females	20%
Selection/culling	EBV
BV estimation method	BLUP animal model
Ratio of missing sire and dam	5%
Heritability of the trait	10%, 25%, or 40%
Phenotypic variance	1.0

**Genome**	

Number of chromosomes	29
Total length	2333 cM
Number of markers	40000/800000
Marker distribution	Evenly spaced
Number of QTL	750
QTL distribution	Random
MAF for markers	0.1
MAF for QTL	0.1
Additive allelic effects for markers	Neutral
Additive allelic effects for QTL	Gamma distribution (shape = 0.40)
Rate of missing marker genotypes	0.01
Rate of marker genotyping error	0.005
Rate of recurrent mutation	0.0001

POP1: population 1; POP2: population 2; EBV: estimated breeding value; BV: breeding value; QTL: quantitative trait loci; MAF: minor allele frequency.

The whole simulation process was repeated 10 times.

The targeted extent and pattern of LD in the simulation was the average of the values reported by [[12] and [13]] for different beef cattle breeds. Detailed information on the extent and pattern of LD were kindly provided by those authors.

#### Genome

The simulated genome consisted of 29 pairs of autosomes with length identical to the real bovine genome based on Btau_3.1 assembling [14] totaling 2333 cM. In most reported simulation studies, just one chromosome was simulated, due to the computing time and memory requirements. The advantage of simulating the real number of autosomes with length identical to the bovine genome is to create a more realistic scenario with respect to the number of physically unlinked marker and QTL loci. The SNP markers were evenly distributed and the initial number of markers was chosen such that it would generate two densities of segregating bi-allelic loci with minor allele frequency (MAF)>0.1: 40 k or 800 k. The markers were neutral in their effect on the trait. A number of QTL was simulated to generate 750 segregating loci with two, three or four alleles and MAF>0.1, whose positions were randomly distributed. Additive allelic effects were randomly sampled from gamma distribution with shape parameter equal to 0.4. The rate of missing marker genotypes was 0.01 and the rate of marker genotyping error was 0.005. A recurrent mutation rate of 10^-5 ^for both markers and QTLs was considered to establish mutation-drift equilibrium in historical generations. The same mutation rate was also applied in all subsequent generations after the historical ones. The parameters used for simulating the genome are given in Table 1 and a summary of the number of simulated SNP markers for each autosome is given in Table 2.

**Table 2:** Average Linkage Disequilibrium (*r^2^*) between adjacent SNP markers.

		POP1 (40 k)		POP2 (800 k)	
**BTA**	**Length** **(Mb)**	**Number** **of** **SNP**	**Average *r^2^*** **(SD)**	**Number** **of** **SNP**	**Average *r^2^*** **(SD)**

1	146	2712	0.25(0.25)	47539	0.33(0.30)
2	126	2205	0.25(0.25)	38629	0.33(0.30)
3	116	2060	0.24(0.25)	38824	0.31(0.29)
4	111	2011	0.23(0.24)	37889	0.30(0.28)
5	119	1749	0.24(0.24)	32230	0.32(0.30)
6	112	1962	0.26(0.26)	38405	0.32(0.29)
7	101	1687	0.25(0.26)	33209	0.31(0.29)
8	104	1748	0.24(0.25)	33833	0.32(0.30)
9	95	1369	0.26(0.26)	30472	0.31(0.29)
10	96	1413	0.27(0.26)	30513	0.32(0.29)
11	102	1551	0.25(0.25)	33513	0.30(0.28)
12	78	1070	0.24(0.26)	23437	0.31(0.29)
13	83	1249	0.25(0.25)	27385	0.32(0.30)
14	82	1290	0.25(0.25)	27452	0.33(0.30)
15	75	1081	0.27(0.27)	22531	0.33(0.30)
16	73	1150	0.24(0.25)	22891	0.33(0.30)
17	70	1195	0.27(0.26)	23281	0.31(0.29)
18	63	987	0.23(0.23)	19391	0.32(0.29)
19	63	966	0.25(0.26)	20719	0.31(0.29)
20	68	1133	0.24(0.24)	21775	0.35(0.31)
21	63	1032	0.25(0.26)	19720	0.32(0.30)
22	60	946	0.26(0.26)	19212	0.31(0.29)
23	49	772	0.25(0.26)	15958	0.31(0.29)
24	60	901	0.24(0.24)	18087	0.32(0.30)
25	42	749	0.25(0.25)	14861	0.31(0.29)
26	48	840	0.25(0.26)	15999	0.30(0.30)
27	43	670	0.26(0.26)	13563	0.32(0.30)
28	40	704	0.25(0.26)	13407	0.32(0.30)
29	45	806	0.26(0.26)	14420	0.33(0.30)

Overall	2333	38008	0.25(0.26)	749145	0.32(0.30)

The results are for each of the 29 autosomes in the recent generations for moderate heritability (0.25) and POP1 (40 k) and POP2 (800 k) over 10 replicates. SD: Standard Deviation.

### Training and prediction sets

The training set was composed of bulls from generations three to eight for both POP1 and POP2. The reason for excluding the first two generations was to use data from a population undergoing selection, which would be mostly the case in beef cattle. In order to estimate the effects of the 40 k segregating markers, all the bulls (n = 1920) that had more than 50 offspring (average progeny size = 73) were selected from POP1 for the three levels of heritability (0.10, 0.25 or 0.40). Of these, 960 and 480 bulls were randomly sampled so that the average accuracy of EBVs was kept the same across the TS sizes within each level of heritability. Therefore, the average progeny size was approximately the same (73) for the three TSs with an average accuracy of EBVs equal to 0.79, 0.90 or 0.94 for heritability of 0.10, 0.25 or 0.40, respectively. For the purpose of estimating the effects of 800 k markers in POP2, only 480 bulls had more than 50 offspring (average progeny size = 73) because of the smaller population size and, therefore, these were selected from POP2 for the three levels of heritabilities (0.10, 0.25 or 0.40). However, considering that this number of bulls with highly accurate EBV (n = 480) is realistic for most beef cattle breeding programs, this scenario would be useful for assessing the accuracy of genomic selection using a high density marker panel.

**Figure 2:**
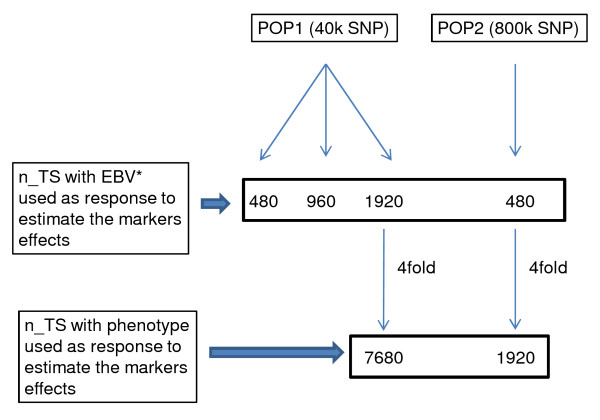
**Schematic representation of the simulated scenarios**. n_TS: number of bulls in the training set. Accuracies of EBV for all n_TS were 0.79, 0.90 or 0.94 for heritability = 0.10, 0.25 or 0.40, respectively. The average progeny size was 73 for all n_TS and heritability levels.

Two other additional TS were created by randomly selecting 7680 and 1920 animals from POP1 and POP2, respectively. In this case, the phenotypes were used to estimate the marker effects, what represented a 4-fold increase in the TS. Figure 2 depicts these simulated scenarios with respect to the TS sizes.

The prediction sets were composed of 8000 and 2000 individuals randomly chosen from the 10^th ^generation of POP1 and POP2, respectively.

### Genetic evaluations

The EBVs of animals were calculated by the standard animal model:

y=1μ+Za+e

where **y **is the vector of trait phenotypes, μ is the overall mean, **Z **is an incidence matrix and **a **is the vector of animals' additive genetic effects and **e **is a vector of random errors. The variance of **y **was assumed to be $ZAZ^{\prime }\sigma_{a}^{2}+I\sigma_{e}^{2}$ where **A **is the additive genetic relationship matrix and $\sigma_{e}^{2}$ is the residual variance.

A ridge regression model was used to estimate SNP effects for using in computing DGEBV.

The model can be written as

$$y=1\mu +X^{\prime }\beta +e$$

where **y **is the vector of either trait phenotypes or EBVs calculated from trait phenotypes and pedigree data by BLUP, μ is the overall mean, **X **is the matrix of marker genotypes for each animal and **β **is the vector of marker effects, and **e **is a vector of random errors.

The marker genotypes of an individual were re-coded as the number of copies of one of the SNP alleles, *i.e*., 0, 1 or 2. The ridge regression factor used assuming a common variance for the marker effects, i.e $\sigma_{a}^{2}{}_{/}\sum_{i=1}^{n}2p_{i}q_{i}$ where n is the number of markers. This is equivalent to GBLUP method proposed by VanRaden [15].

The DGEBV was computed for the animals in the prediction set as

$$\hat{a}=X^{\prime }\hat{\beta }$$

where **X **is the matrix of marker genotypes for each animal in the prediction set and $\hat{\beta }$ is the vector of estimated marker effects.

The gebv software [16] was used to estimate marker effects and DGEBV.

The accuracy of DGEBV was calculated as the correlation between the DGEBV and the true breeding value and standard errors were computed as the standard deviation of the accuracies across the 10 replicates, divided by $\sqrt{10}$.

The differences between the average accuracies of DGEBV across scenarios were tested by t-test.

The accuracies of DGEBV were regressed on number of animals in the TS, using a quadratic regression, to assess the effect of increasing the TS size.

### Linkage Disequilibrium

Linkage Disequilibrium was measured by *r^2^*, which is the squared correlation of the alleles at 2 loci [17]:

$$r^{2}=\frac{{\text{D}}^{\text{2}}}{\text{f}\left(\text{A}\right)\text{f}\left(\text{a}\right)\text{f}\left(\text{B}\right)\text{f}\left(\text{b}\right)}$$

where D = f(AB)-f(A)f(B), and f(AB), f(A), f(a), f(B), f(b) are observed frequencies of haplotypes AB and of alleles A, a, B, b, respectively.

It was demonstrated that *r^2 ^*is a more suitable measure to estimate LD for biallelic markers, such as SNPs, because *r^2 ^*is less sensitive to allelic frequency and sample size than D' [18-20].

Due to the computing time required to calculate all possible pair-wise LD, the LD statistics for all pair-wise LD were calculated only for BTA 1 and for one replicate. However, the statistics should illustrate well the decay of simulated LD for the entire genome. The statistics for LD between all adjacent SNP were calculated, however, for all 29 BTA.

## Results and Discussion

### Linkage disequilibrium

The average levels of LD between adjacent SNP in the recent generations of POP1 and POP2 are given in Table 2. Table 3 presents the average LD for different distances between all closely located SNP pairs (<1 Mb) in bins of 0.1 Mb for BTA1.

**Table 3:** Average Linkage Disequilibrium (*r^2^*) for different distances between closely located SNP pairs.

		POP1 (40 k)			POP2 (800 k)		
		
h^2^	Distance range (Mb)	Pairs	*r^2 ^*(SD)	Frequency *r2 *> 0.30 (%)	Pairs	*r^2 ^*(SD)	Frequency *r2 *> 0.30 (%)
	0.00-0.10	5049	0.23 (0.24)	1512 (29.95)	4275	0.24 (0.25)	1272 (29.75)
	0.10-0.20	5074	0.17 (0.19)	1063 (20.95)	4474	0.18 (0.20)	919 (20.54)
	0.20-0.30	6265	0.14 (0.16)	885 (14.13)	4290	0.14 (0.16)	644 (15.01)
	0.30-0.40	4938	0.12 (0.14)	510 (10.33)	4358	0.12 (0.14)	493 (11.31)
	0.40-0.50	5042	0.10 (0.13)	425 (8.43)	4280	0.10 (0.13)	359 (8.39)
0.10	0.50-0.60	6199	0.09 (0.11)	370 (5.97)	4196	0.09 (0.12)	279 (6.65)
	0.60-0.70	4994	0.08 (0.10)	228 (4.57)	4208	0.09 (0.11)	242 (5.75)
	0.70-0.80	5975	0.07 (0.09)	187 (3.69)	4268	0.08 (0.10)	190 (4.45)
	0.80-0.90	6183	0.07 (0.09)	167 (2.70)	4334	0.07 (0.10)	170 (3.92)
	0.90-1.00	4917	0.06 (0.08)	128 (2.60)	4163	0.07 (0.09)	149 (3.58)
	
	Overall	3932610	0.008 (0.03)	6151 (0.16)	3126250	0.01 (0.03)	6062 (0.19)

	0.00-0.10	5248	0.22 (0.24)	1495 (28.49)	4494	0.24 (0.25)	1375 (30.60)
	0.10-0.20	5224	0.17 (0.19)	1077 (20.62)	4740	0.18 (0.20)	1006 (21.22)
	0.20-0.30	6528	0.13 (0.16)	855 (13.10)	4630	0.14 (0.17)	724 (15.64)
	0.30-0.40	5147	0.11 (0.14)	461 (8.96)	4560	0.12 (0.14)	488 (10.70)
	0.40-0.50	5112	0.09 (0.12)	315 (6.16)	4596	0.11 (0.13)	406 (8.83)
0.25	0.50-0.60	6466	0.08 (0.11)	343 (5.31)	4469	0.10 (0.12)	333 (7.45)
	0.60-0.70	5146	0.07 (0.09)	186 (3.61)	4418	0.09 (0.11)	250 (5.66)
	0.70-0.80	5227	0.07 (0.09)	139 (2.66)	4502	0.08 (0.10)	226 (5.02)
	0.80-0.90	6431	0.06 (0.08)	178 (2.77)	4359	0.08 (0.10)	207 (4.75)
	0.90-1.00	5129	0.06 (0.08)	95 (1.85)	4416	0.07 (0.09)	154 (3.49)
	
	Overall	4096952	0.006 (0.03)	5689 (0.14)	3283438	0.011 (0.03)	6543 (0.20)

	0.00-0.10	5439	0.22 (0.24)	1517 (27.89)	4191	0.24 (0.24)	1265 (30.18)
	0.10-0.20	5468	0.17 (0.19)	1070 (19.57)	4225	0.17 (0.20)	865 (20.47)
	0.20-0.30	6801	0.13 (0.16)	886 (13.03)	4263	0.14 (0.16)	621 (14.57)
	0.30-0.40	5435	0.11 (0.14)	543 (9.99)	4251	0.12 (0.14)	457 (10.75)
	0.40-0.50	5444	0.09 (0.12)	361 (6.63)	4286	0.10 (0.12)	333 (7.77)
0.40	0.50-0.60	6782	0.08 (0.10)	293 (4.32)	4284	0.09 (0.11)	270 (6.30)
	0.60-0.70	5377	0.07 (0.09)	176 (3.27)	4103	0.09 (0.11)	232 (5.65)
	0.70-0.80	5374	0.07 (0.09)	150 (2.79)	4260	0.08 (0.10)	205 (4.81)
	0.80-0.90	6733	0.06 (0.08)	155 (2.30)	4285	0.07 (0.09)	136 (3.17)
	0.90-1.00	5342	0.06 (0.07)	75 (1.40)	4199	0.06 (0.08)	104 (2.48)
	
	Overall	4290985	0.006 (0.03)	5710 (0.13)	3072154	0.010 (0.03)	5305 (0.17)

The results are for chromosome 1 in the recent generations for three heritability levels and POP1 (40 k) and POP2 (800 k) for one replicate. For POP2 (800 k), 40 k markers were randomly sampled to represent the 800 k panel.

The overall *r^2 ^*between adjacent SNP across all autosomes for moderate heritability (0.25) in the recent generations was 0.25 and 0.32 for POP1 (40 k) and POP2 (800 k), respectively (Table 2). This average *r^2 ^*between adjacent SNPs for POP1 is similar to the value reported by [21] for Gyr cattle in Brazil (*r^2^*= 0.21), using bulls genotyped with the Illumina BovineSNP50 chip and similar to the *r^2 ^*reported by [12] for a multi-breed herd genotyped with the Illumina BovineSNP50 chip in Canada (*r^2 ^*= 0.21 between markers 30-35 kb apart).

Table 3 shows the average *r^2 ^*between all SNP pairs for different distance ranges up to 1 Mb for BTA1. The highest average estimated *r^2 ^*was in the range of 0.22-0.24 for a distance of 0-0.10 Mb. The *r^2 ^*values decreased with increasing distance between SNP, but not as sharply as the decrease reported for Holstein cattle [22]. The trend of decay of LD with the increase in physical distance was, as expected, exponential for both POP1 and POP2 (Table 3) in agreement with other published studies [21-23].

In the current simulation study, for moderate heritability (0.25) and POP1 (40 k), the % of *r^2 ^*> 0.30 for ranges 0-0.1 Mb, 0-0.2 Mb, 0-0.5 Mb and 0-1 Mb was 28%, 25%, 15% and 9%, respectively. These results are similar to findings of [21] for Gyr cattle, who reported a % of *r^2 ^*> 0.30 of 23%, 20%, 14% and 10% for the same ranges, respectively.

Using the Illumina BovineSNP50 chip, [13] reported *r^2 ^*of 0.31, 0.22 and 0.15 for Angus and crossbred cattle for distance ranges between 0-0.03 Mb, 0.03-0.06 Mb and 0.06-0.10 Mb, respectively. The simulated results in the present study for the distance range 0-0.10 Mb seem in line with those results reported by [13].

Mckay et al. [24] reported the *r^2 ^*for eight breeds of cattle varying from 0.15 to 0.20 for a distance range from 0-0.1 Mb, which is similar to the r^2 ^observed in the simulated data for the same distance range (Table 3).

Table 4 shows the average LD (*r^2^*) between adjacent SNPs and distribution of SNP pairs across different LD ranges on chromosome 1 in the recent generations for three heritability levels and POP1 and POP2. The % of SNP pairs with *r^2 ^*> 0.30 did not differ across the heritabilities, probably because the number of generations under selection was small (10).

**Table 4:** Average Linkage Disequilibrium (*r^2^*) between adjacent SNPs pairs and distribution across different *r^2 ^*ranges.

		Number of SNP Pairs and (%)		
	LD (r^2^) range	0.10	0.25	0.40
	
POP1 (40 K)	0.00-0.10	975 (36.97)	1040 (38.35)	1060 (37.80)
	0.10-0.20	463 (17.56)	468 (17.26)	501 (17.87)
	0.20-0.30	305 (11.57)	316 (11.65)	331 (11.80)
	0.30-0.40	232 (8.80)	232 (8.55)	248 (8.84)
	0.40-0.50	192 (7.28)	195 (7.19)	182 (6.49)
	0.50-0.60	126 (4.78)	142 (5.24)	136 (4.85)
	0.60-0.70	110 (4.17)	98 (3.61)	107 (3.82)
	0.70-0.80	83 (3.15)	78 (2.88)	81 (2.89)
	0.80-0.90	62 (2.35)	66 (2.43)	79 (2.82)
	0.90-1.00	89 (3.38)	77 (2.84)	79 (2.82)
	
	Average LD (r2)	0.26	0.25	0.25
POP2 (800 K)	0.00-0.10	13980 (29.54)	14124 (29.71)	14939 (31.03)
	0.10-0.20	8034 (16.98)	7443 (15.66)	8072 (16.77)
	0.20-0.30	5388 (11.38)	5442 (11.45)	5571 (11.57)
	0.30-0.40	4149 (8.77)	4240 (8.92)	4156 (8.63)
	0.40-0.50	3250 (6.87)	3418 (7.19)	3321 (6.90)
	0.50-0.60	2859 (6.04)	2837 (5.97)	2665 (5.54)
	0.60-0.70	2359 (4.98)	2479 (5.21)	2274 (4.72)
	0.70-0.80	1928 (4.07)	2162 (4.55)	2121 (4.41)
	0.80-0.90	1929 (4.08)	2032 (4.27)	1924 (4.00)
	0.90-1.00	3451 (7.29)	3362 (7.07)	3095 (6.43)
	
	Average LD (r2)	0.33	0.33	0.32

The results are for chromosome 1 in the recent generations for three heritability levels and POP1 (40 k) and POP2 (800 k) over 10 replicates.

When comparing the marker density, 43% of SNP pairs showed high LD (*r^2 ^*> 0.30) in POP2 (800 k) while 33% of the SNP pairs showed high LD in POP1 (40 k) for moderate heritability (0.25). This difference of ten points seems not very high, but when it is translated to number of SNP pairs in high LD in each marker density, it becomes quite significant and has an impact on the accuracy of genomic selection, as shown later in the results.

### Accuracy of DGEBV

Overall, the accuracy of DGEBV increased significantly (p < 0.05) with the increase in the number of bulls in the TS, heritability of the trait and density of markers (Table 5). When the EBV was used to estimate the marker effects in POP1 (40 k), the number of bulls in the TS, which varied from 480 to 1920, increased the accuracy of DGEBV for all three heritability levels.

**Table 5:** Accuracy of direct genomic estimated breeding value.

			POP1(40 k)			POP2(800 k)	
	**h^2^**	**480**	**960**	**1920**	**7680**	**480**	**1920**

	0.10	-	-	-	0.44a,a	-	0.30b,a
PHE	0.25	-	-	-	0.56a,b	-	0.41b,b
	0.40	-	-	-	0.65a,c	-	0.50b,c

	0.10	0.37a,a	0.45b,a	0.56c,a	-	0.43b,a	-
EBV	0.25	0.37a,a	0.49b,b	0.60c,b	-	0.46d,ab	-
	0.40	0.39a,b	0.51b,c	0.64c,c	-	0.48d,b	-

The accuracies of Direct Genomic Estimated Breeding Value (DGEBV) are for animals in the prediction set, considering two levels of marker densities, different numbers of bulls in the training set (TS), three heritability levels, and two alternate response variables used for calculating the marker effects - phenotype (PHE) or Estimated Breeding Value (EBV). The accuracy of EBV used for calculating the marker effects was 0.79, 0.90 or 0.94 for the heritability 0.10, 0.25 or 0.40, respectively, regardless the population and number of bulls in the TS. The average progeny size was 73 for all TS sizes, except for those were the phenotypic record was used to estimate the marker effects. The results are presented as the average over 10 replicates. Different letters indicate significant differences (p < 0.05) by t-test (the first letter indicates differences within rows, while the second letter indicates differences within columns).

**Figure 3:**
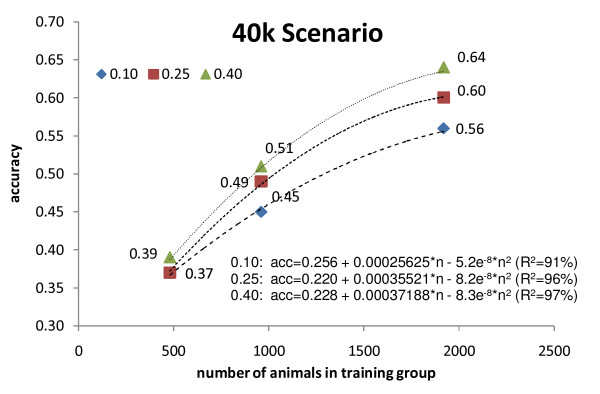
**Accuracy of Direct Genomic Estimated Breeding Value (DGEBV) for 40 k Scenario**. DGEBV as a function of number of animals in the training set, considering three levels of heritability and 40 k markers. The results are presented as the average of 10 replicates.

The DGEBV accuracies were regressed on the number of bulls in the TS, using a quadratic regression. The estimated regressions were y = 0.256 + 0.00026x - 5.2e^-8^x^2 ^(p < 0.0001; R^2 ^= 0.91), y = 0.220 + 0.00036x - 8.2e^-8^x^2 ^(p < 0.0001; R^2 ^= 0.96) and y = 0.228 + 0.00037x - 8.3e^-8^x^2 ^(p < 0.0001; R^2 ^= 0.97) for heritability = 0.10, 0.25 and 0.40, respectively. Figure 3 depicts the estimated regressions for each heritability level. It is expected that the accuracy of DGEBV would increase 0.15, 0.20 and 0.21 points for an increase from 480 to 1480 bulls in the TS, and 0.11, 0.12 and 0.14 points from 920 to 1920 bulls in the TS, for heritability of 0.10, 0.25 and 0.40 respectively. These results do not fully agree with those from [3], who used Holstein bulls genotyped for the Illumina BovineSNP50 chip and reported that gains from genomic data increased almost linearly with number of bulls in the TS. Although it not possible to extrapolate out of the range of number of bulls in the TS, the current results suggest that it will be necessary genotyping a large number of bulls to obtain large gains with genomic selection, and a plateau might be expected, limiting the possible gain in accuracy of DGEBV for the density of markers considered (40 k) and the extent of LD simulated.

The highest accuracy (0.64) was observed for heritability of 0.40 and 1920 bulls in the TS (Table 5). This value implies that 41% of genetic variance of the trait was explained by marker effects. These results are similar to those reported by [8], using data from 2000 Angus bulls. The author reported correlations from 0.5 to 0.7 between conventional EBV and genomic predictions. The author, however, did not report the accuracy of conventional EBV used to calculate the correlations.

The effect of heritability was not as evident for 480 bulls in the TS, as it was for 960 and 1920 bulls (Table 5), reflecting the fact that the sample size became a more limiting factor than the heritability level in the TS of 480 bulls.

The response variable used for calculating the marker effects also affected the accuracy of DGEBV. Table 5 shows that when the phenotypes were used as response variable to estimate marker effects in POP1 (40 k), but with a 4-fold TS size (n = 7680), the accuracy of DGEBV was higher than that from using EBVs from 480 bulls for all heritability levels (p < 0.05). However, when EBVs from 1920 bulls instead of own phenotypes from 7680 bulls were used to estimate the marker effects, the accuracy of DGEBV was higher for heritabilities of 0.10 and 0.25 (p < 0.05). Therefore, in this study, genotyping 4-fold more animals (7680 vs. 1920) and using own phenotypes instead of EBVs to estimate the marker effects was not sufficient to increase the accuracy of DGEBV for moderate to low heritability traits.

Table 5 also presents the DGEBV accuracy for the 800 k markers scenario. Because of the computing time and memory requirements, it was possible to simulate only 480 bulls with accurate EBV in the TS. The estimated accuracies of DGEBV were 0.43, 0.46 and 0.48 for heritability 0.10, 0.25 and 0.40, respectively. The accuracies of DGEBV in this scenario were higher (p < 0.05) than those from 40 k SNP markers and 480 bulls in the TS and similar to those with 40 k SNP markers and 960 bulls in the TS. These results indicate that the higher level of LD between the 800 k SNP markers would require 2 times less animals in the TS. Muir [25], using simulated data, reported that increasing number of markers had contradictory effects on accuracy of GEBV, because of co-linearity between the effects of markers. Then it might be expected that the 800 k SNP scenario would benefit more from an increase in the TS size and differences with respect to the accuracy of DGEBV from the 40 k SNP scenario would increase.

When the number of bulls genotyped with 800 k SNP markers was 1920 and their phenotypes were used for estimating marker effects, the accuracy of DGEBV increased only for heritability 0.40 (p < 0.10), while for the other two levels of heritability the accuracy decreased (p < 0.05) compared to accuracies from 480 bulls in the TS and using EBV for estimating marker effects.

One of the main advantages of using genomic information is for traits difficult or expensive to measure for which traditional evaluations are not usually available or to obtain accurate EBV early in the animal's life, when the own phenotypic information has not been measured yet. For the latter situation, if we consider that the average EBV of the parents is the only available information early in the animal's life, one could compare the accuracies provided by PA and the accuracies of DGEBV. Since the reliability of PA is a quarter of the sum of reliabilities of the EBV of parents, considering the average reliability of EBV of bulls in the TS reported in this study and the average reliability of the EBVs of the dams in the simulated data, the expected accuracies for PA would be 0.44, 0.53 and 0.58 for heritability = 0.10, 0.25 and 0.40, respectively. These accuracies are smaller than those accuracies of DGEBV considering 1920 bulls in the TS for POP1 (40 k) for all heritability levels. Therefore, the gains in accuracy by using DGEBV would be of 27%, 13% and 10% compared to accuracy of PA for these conditions. If PA is unavailable, then the use of DGEBV could potentially be even more valuable.

Schenkel et al. [4] reported results from a large genomic prediction validation study in Canada for 44 traits of dairy cattle, using 6403 bulls in the TS genotyped for the Illumina BovineSNP50 chip. The genomic predictions showed an increase of reliability for 42 of the 44 traits. For production traits the average gain in reliability was of 41%, using domestic proofs only and 74%, using both domestic and MACE proofs. Therefore, as expected, the gains reported in the current study that tried to mimic the LD structure that one might expect in a beef cattle population were lower than those reported in dairy cattle.

The main issue that has been discussed in most studies involving genomic selection is whether it will bring additional benefits, considering the cost of genotyping. An alternative to reduce the cost of genotyping and, thus, increase the number of genotyped animals, is the imputation of genotypes, which would allow genotyped animals with a less dense SNP panels (e.g., 3 k) to be imputed to a denser SNP panels (e.g., 50 k), using a reference population genotyped with the denser SNP panel [26]. This alternative should be considered primordial in the projects that involve genomic selection, mainly in countries where the number of available bulls with accurate EBV for genotyping is much smaller than those considered in this study. In this way, with the use of imputation, more bulls, even those with less accurate EBV, could be incorporated to the TS. Collaboration among institutions involved in genomic selection research and application in beef cattle through the sharing genotypes could also increase the TS sizes, which would make more feasible the incorporation of genomic information in breeding programs.

## Conclusions

As reported in dairy cattle, the size of the training set and the extent of linkage disequilibrium have major impact on the accuracy of direct genomic EBV. Based on the findings of this simulation study, large training sets, as well as dense marker panels, aiming to increase the level of linkage disequilibrium between markers and QTL, will likely be needed in beef cattle for successful implementation of genomic selection.

Gains in accuracy by using direct genomic EBV and, therefore, before the animal having an accurate EBV, showed more advantage for traits with a moderate to low heritability.

Increasing the number of animals in the TS by 4-fold and using their phenotypes to estimate marker effects was not sufficient to maintain or increase the accuracy of direct genomic EBV obtained using estimated breeding values when the trait heritability was lower than 40% for both marker densities, i.e. 40 k and 800 k SNPs.

This investigation provides preliminary insights on expected gains in accuracy by using direct genomic EBV in beef cattle. Additional simulation studies are warranted.

## Competing interests

The authors declare that they have no competing interests.

## Authors' contributions

FVB carried out the simulations, performed the simulated data analysis and drafted the manuscript. MS participated in the design of the simulations process and wrote the code of the main software used in the study. JBN and JAC critically revised the manuscript. FSS contributed to the design of the simulations and analysis of simulated data. In addition, FSS revised the manuscript. All authors read and approved the final manuscript.
